# Supplementary material for: RNA targeting unleashes indiscriminate nuclease activity of CRISPR–Cas12a2
Source: Nature. 2023 Jan 4;613(7944):582–7. doi: 10.1038/s41586-022-05560-w (PMC9849127; doi:10.1038/s41586-022-05560-w)
Supplement: Supplementary file 1 — This file contains Supplementary Fig. 1 and Table 1 and the legend to Supplementary Video 1. [file 41586_2022_5560_MOESM1_ESM.pdf]

nature portfolio

https://doi.org/10.1038/s41586-022-05560-w

Supplementary information

RNA targeting unleashes indiscriminate nuclease activity of CRISPR–Cas12a2

In the format provided by the authors and unedited

Supplementary Information

RNA targeting unleashes indiscriminate nuclease activity of CRISPR-Cas12a2

Jack P. K. Bravo, Thomson Hallmark, Bronson Naegle, Chase L. Beisel, Ryan N. Jackson, David W. Taylor

Table of Contents

Supplementary Figure 1 l Source data. Raw data for all gels. 1

Supplementary Table 1 l Strains, plasmids, and nucleic acid substrates. 4

Supplementary Video 1 l Structural rearrangements in Cas12a2. Cas12a2 binary complex coloured as in **Fig. 1**. Morph to ternary complex, upon binding of target strand (orange). Rotation showing the crRNA:TS duplex running through the centre of the complex. RuvC active site, with collateral dsDNA bound (purple and green). Zoom-in on the two aromatic clamp pairs (non-cleaved strand and then cleaved strand).

Figure 1. Source data. Raw data for all gels.

<u>Raw data for Fig. 2g.</u>

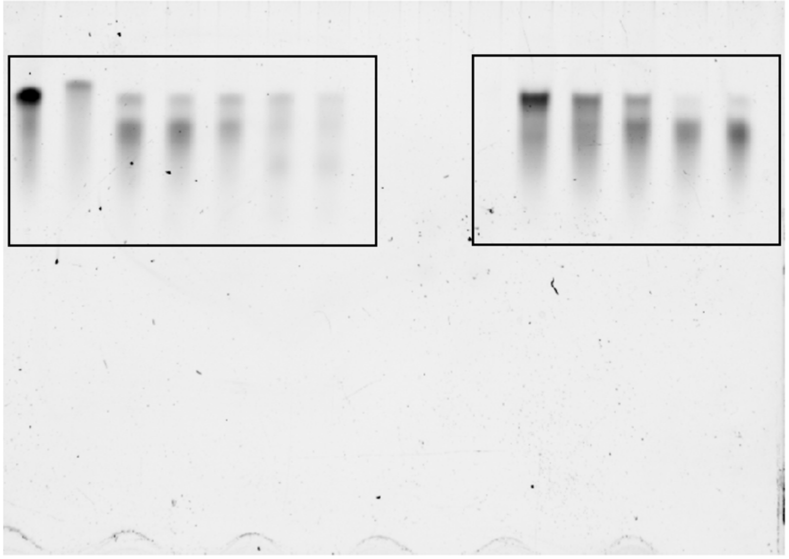

Sybr gold staining

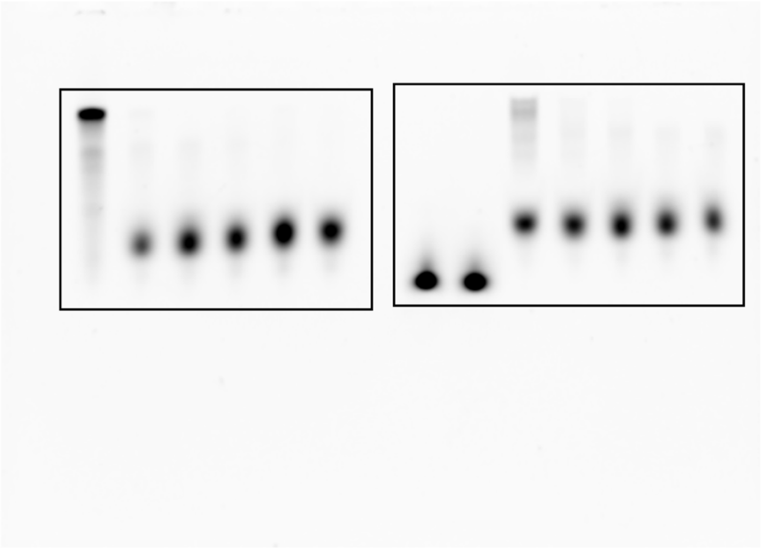

Fluorescence from FAM label

Raw data for Fig. 3h.

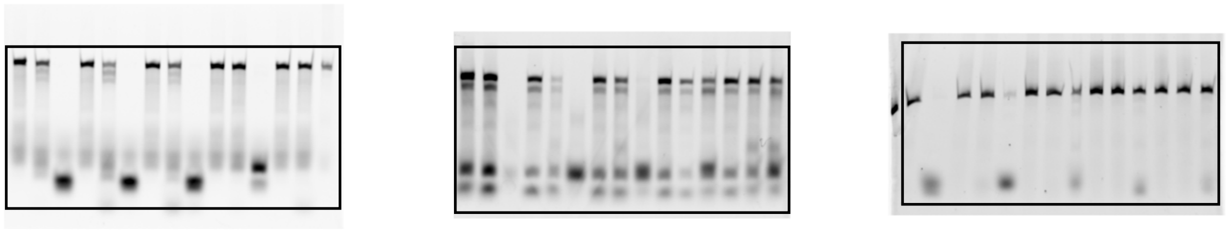

Left to right. ssDNA, ssRNA, dsDNA.

Raw data for Extended Data Fig. 4e.

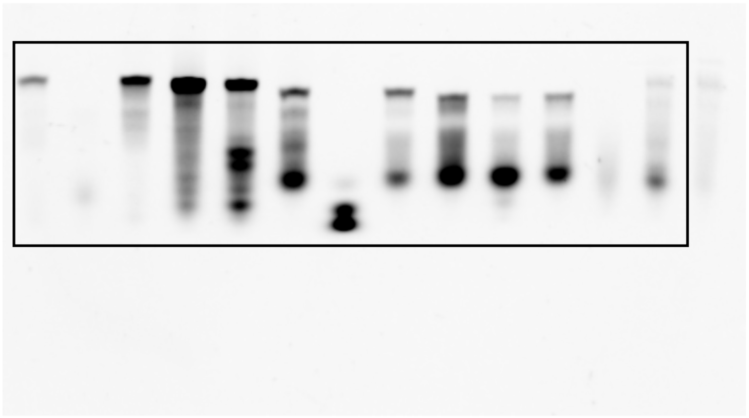

Raw data for Extended Fig. 8b.

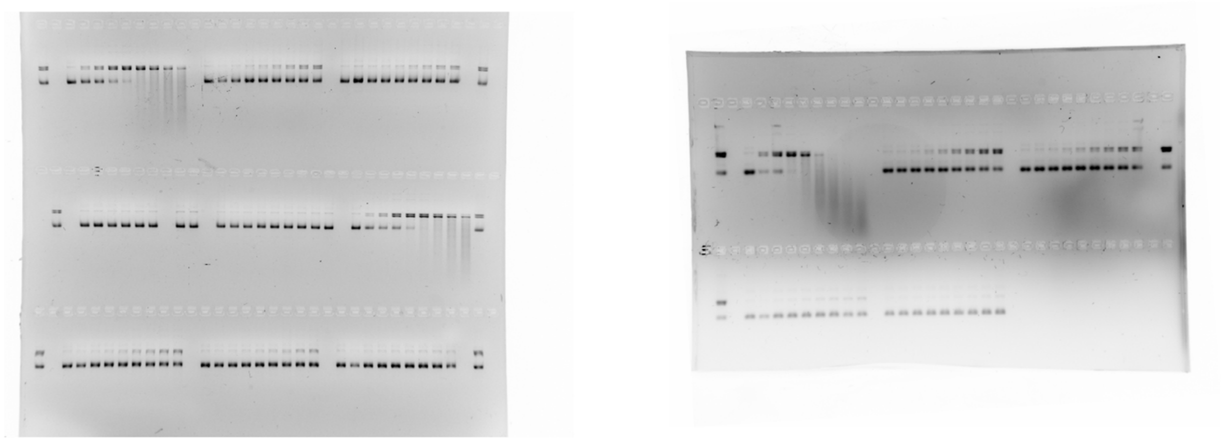

Raw data for Extended Data Fig. 9.

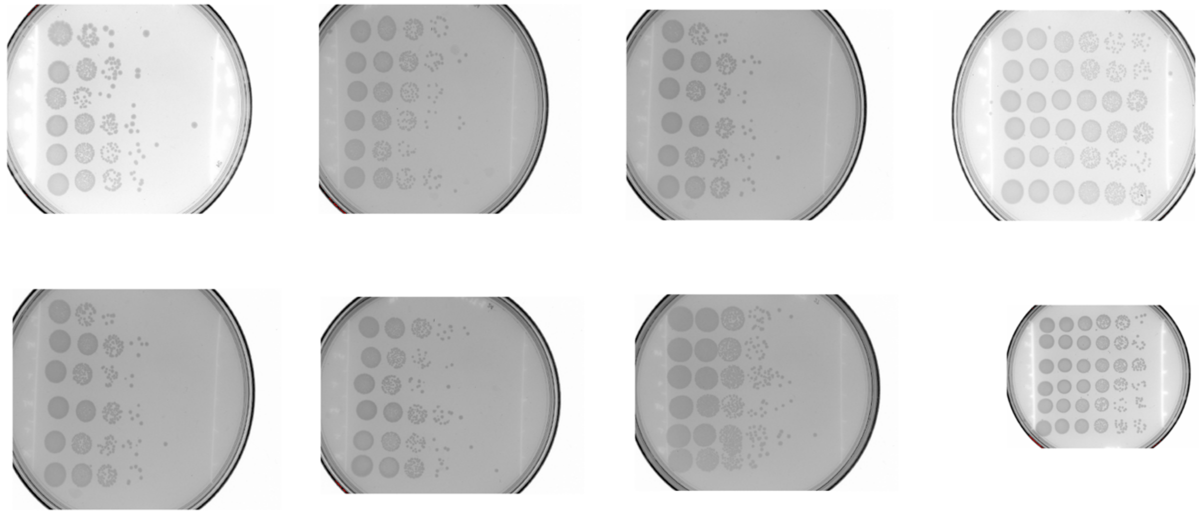

Top (left to right). E1063A, Y465A, Y1080A, Y465A/Y1080A
Bottom (left to right). Y1069A, F1092A, Y1069A/F1092A, ΔPI

Table 1. Strains, plasmids, and nucleic acid substrates.

Substrate	Abrv.	Sequence	FAM
**Biochemistry**			
crRNA	CR	5′-UAAAUUUCUACUAUUGUAGAUUGGAGCAACACCUGAAGGAAGGCUUGA-3’	
Target Strand	TS	5′-UCUUUGCUGCCGCACUUGCUCAUCAAGCCU,UCCUU,CAGGUGUUGCUCCAGAAAGGUGAGUUCUUCUUGUUGUU-3’	5′
Target Strand ΔPFS	TSΔPFS	5′-UCUUUGCUGCCGCACUUGCUCAUCAAGCCUUCCUUCAGGUGUUGCUCCA-3′	5′
Target Strand -5	TS-5	5′-UCCUUCAGGUGUUGCUCCAGAAAGGUGAGUUCUUCUUGUUGUU-3′	3′
Target Strand -10	TS-10	5′-CAGGUGUUGCUCCAGAAAGGUGAGUUCUUCUUGUUGUU-3′	3′
Target Strand -9	TS-9	5′-UCAGGUGUUGCUCCAGAAAGGUGAGUUCUUCUUGUUGUU-3′	3′
Target Strand -8	TS-8	5′-UUCAGGUGUUGCUCCAGAAAGGUGAGUUCUUCUUGUUGUU-3′	3′
Nontarget RNA	NT-RNA	5′-CAGAGAUAAGUGACGCGCGGCGAGUGGCGCGCCACGUCGGAAAUCUAGAGGCG-3′	5′
Nontarget ssDNA	NT-ssDNA	5′-AACTGATATGACAATTGCGCGTAGCACGACGACGATATGACACTTGCGCATAACGACGACGATACAATGAT-3′	5′
Nontarget dsDNA	NT-dsDNA	5′-AACTGATATGACAATTGCGCGTAGCA<br>3′-TTGACTATACTGTTAACGCGCATCGT	5′

		CGACGACGATATGACACTTGCGCATAAC<br>GCTGCTGCTATACTGTGAACGCGTATTG<br><br>GACGACGATACAATGAT-3′<br>CTGCTGCTATGTTACTA-5′	
**Structure**			
crRNA			None
Target Strand			None
Target Strand - flanks	TS-F		
PT Nontarget dsDNA	PT-dsDNA		None
**Plasmids**			
1408	pACYC_SuCas12a2_3xcrRNA		
1416	p___SuCas12a2		
dPI mutant 2290	pACYC_SuCas12a2(Δa.a. 624 - 713)_3xcrRNA		
2286	pACYC_SuCas12a2(Y465A)_3xcrRNA		
2287	pACYC_SuCas12a2(Y1080A)_3xcrRNA		
2379	pACYC_SuCas12a2(Y465A, Y1080A)_3xcrRNA		

2247	pACYC_SuCas12a2(Y1069A)_3xcrRNA		
2288	pACYC_SuCas12a2(F1092A)_3xcrRNA		
2289	pACYC_SuCas12a2(Y1069A, F1092A)_3xcrRNA		
2270	pACYC_SuCas12a2(R1180A)_3xcrRNA		
2271	pACYC_SuCas12a2(D1184A)_3xcrRNA		
1412	pACYC_SuCas12a2(E1063A)_3xcrRNA		
Target(1094)	pET27b(+)_Target_TTTC		
Non-target(1095)	pET27b(+)_non-Target_TTTC		
**Primers**			
Cas12a2(Y465A)F		AGAAATCTTAgcTGGTAACACGATGTTC	
Cas12a2(Y465A)R		CAATTTGAAACTTATAAGAAGGATATAC	
Cas12a2(Y1080A)F		TGATGGCAGAgcCCTTCCGTCATAAC	
Cas12a2(Y1080A)R		ACTTCACAAGACGAGGATG	
Cas12a2(Y1069A)F		TGCACCTCCAgcGGCTCTTGAC	
Cas12a2(Y1069A)R		AAGTCCGGTTATGACGGA	
Cas12a2(F1092A)F		CTGTTCCTTGgcATCTACATCCTC	
Cas12a2(F1092A)R		CAGAATCAGATGCTTGCAG	

Cas12a2(R1180A)F		AAATGTATACgcGGACAAGGATGACATCTTG	
Cas12a2(R1180A)R		GTTTTGCTACAGACGGGG	
Cas12a2(D1184A)F		GACAAGGATGcCATCTTGGTTTG	
Cas12a2(D1184A)R		CCTGTATACATTTGTTTTGC	
Cas12a2(Δa.a.624 - 713)F	dPI	GCTTGTAAGGCTCTTAAC	
Cas12a2(Δa.a.624 - 713)R	dPI	TCATATTTGCTGCAGAGC	
