# Supplementary material for: RNA targeting unleashes indiscriminate nuclease activity of CRISPR–Cas12a2
Source: Nature. 2023 Jan 4;613(7944):582–7. doi: 10.1038/s41586-022-05560-w (PMC9849127; doi:10.1038/s41586-022-05560-w)
Supplement: Supplementary file 2 — Reporting Summary [file 41586_2022_5560_MOESM2_ESM.pdf]

nature portfolio

Corresponding author(s): Ryan N. Jackson and David W. Taylor

Last updated by author(s): Nov 4, 2022

# Reporting Summary

Nature Portfolio wishes to improve the reproducibility of the work that we publish. This form provides structure for consistency and transparency in reporting. For further information on Nature Portfolio policies, see our Editorial Policies and the Editorial Policy Checklist.

## Statistics

For all statistical analyses, confirm that the following items are present in the figure legend, table legend, main text, or Methods section.

| n/a | Confirmed | |
|---|---|---|
| ☐ | ☒ | The exact sample size ($n$) for each experimental group/condition, given as a discrete number and unit of measurement |
| ☐ | ☒ | A statement on whether measurements were taken from distinct samples or whether the same sample was measured repeatedly |
| ☐ | ☒ | The statistical test(s) used AND whether they are one- or two-sided<br>*Only common tests should be described solely by name; describe more complex techniques in the Methods section.* |
| ☒ | ☐ | A description of all covariates tested |
| ☒ | ☐ | A description of any assumptions or corrections, such as tests of normality and adjustment for multiple comparisons |
| ☐ | ☒ | A full description of the statistical parameters including central tendency (e.g. means) or other basic estimates (e.g. regression coefficient) AND variation (e.g. standard deviation) or associated estimates of uncertainty (e.g. confidence intervals) |
| ☐ | ☒ | For null hypothesis testing, the test statistic (e.g. $F$, $t$, $r$) with confidence intervals, effect sizes, degrees of freedom and $P$ value noted<br>*Give P values as exact values whenever suitable.* |
| ☒ | ☐ | For Bayesian analysis, information on the choice of priors and Markov chain Monte Carlo settings |
| ☒ | ☐ | For hierarchical and complex designs, identification of the appropriate level for tests and full reporting of outcomes |
| ☒ | ☐ | Estimates of effect sizes (e.g. Cohen's $d$, Pearson's $r$), indicating how they were calculated |

*Our web collection on statistics for biologists contains articles on many of the points above.*

## Software and code

Policy information about availability of computer code

| | |
|---|---|
| Data collection | Data were collected on a FEI Glacios cryo-TEM equipped with a Falcon 4 detector. Data was collected in SerialEM v3.8, with a pixel size of 0.94 Å, a defocus range of -1.5 - -2.5 µm, and a total exposure time of 15s resulting in a total accumulated dose of 40 e/Å2 which was split into 60 EER fractions. |
| Data analysis | Motion correction, CTF estimation and particle picking was performed on-the-fly using cryoSPARC Live v4.0.0-privatebeta.2 and cryoSPARC v3.2. All subsequent data processing was performed in cryoSPARC v3.2. Models were built using COOT v1.0 and relaxed using flexible molecular dynamics fitting with ISOLDE v1.4, then finally subjected to real-space refinement as implemented in PHENIX v1.19.2. All structural figures and movies were generated using ChimeraX v1.0 , apart from the modevectors, which were generated in PyMol v2.5. |

For manuscripts utilizing custom algorithms or software that are central to the research but not yet described in published literature, software must be made available to editors and reviewers. We strongly encourage code deposition in a community repository (e.g. GitHub). See the Nature Portfolio guidelines for submitting code & software for further information.

# Data

Policy information about availability of data

All manuscripts must include a data availability statement. This statement should provide the following information, where applicable:

- Accession codes, unique identifiers, or web links for publicly available datasets
- A description of any restrictions on data availability
- For clinical datasets or third party data, please ensure that the statement adheres to our policy

The atomic models of Cas12a2 binary, tertiary and quaternary complexes have been deposited into the Protein Data Bank (PDB) with codes PDB 8D49, PDB 8D48 and PDB 8D4A, and the corresponding maps have been deposited into the Electron Microscopy Data Bank (EMDB) with codes EMD-29178, EMD-27180 and EMD-27179, respectively. Requests for materials can be sent to R.N.J. (ryan.jackson@usu.edu) and D.W.T. (dtaylor@utexas.edu).

# Human research participants

Policy information about studies involving human research participants and Sex and Gender in Research.

| | |
|---|---|
| Reporting on sex and gender | N/A |
| Population characteristics | N/A |
| Recruitment | N/A |
| Ethics oversight | N/A |

Note that full information on the approval of the study protocol must also be provided in the manuscript.

# Field-specific reporting

Please select the one below that is the best fit for your research. If you are not sure, read the appropriate sections before making your selection.

☒ Life sciences ☐ Behavioural & social sciences ☐ Ecological, evolutionary & environmental sciences

For a reference copy of the document with all sections, see nature.com/documents/nr-reporting-summary-flat.pdf

# Life sciences study design

All studies must disclose on these points even when the disclosure is negative.

| | |
|---|---|
| Sample size | A total of ~2000 micrographs were collected for each sample. Each data set contained ~1 million particles. These are typical numbers for cryo-EM data sets to obtain high-resolution structures and provided the required resolution required for our structural analysis and results. |
| Data exclusions | 2D and 3D classification were used to remove damaged particles. This is standard practice in cryo-EM studies and is necessary to obtain homogenous particles for reconstruction. |
| Replication | Cryo-EM datasets were collected on multiple samples in separate imaging sessions with identical overall domain structures. |
| Randomization | No randomization was performed. This study does not require randomization because no animal or human subjects were used. |
| Blinding | No blinding was performed. This study does not require blinding because no animal or human subjects were used. |

# Reporting for specific materials, systems and methods

We require information from authors about some types of materials, experimental systems and methods used in many studies. Here, indicate whether each material, system or method listed is relevant to your study. If you are not sure if a list item applies to your research, read the appropriate section before selecting a response.

## Materials & experimental systems

| n/a | Involved in the study |
| --- | --- |
| ☒ | ☐ Antibodies |
| ☒ | ☐ Eukaryotic cell lines |
| ☒ | ☐ Palaeontology and archaeology |
| ☒ | ☐ Animals and other organisms |
| ☒ | ☐ Clinical data |
| ☒ | ☐ Dual use research of concern |

## Methods

| n/a | Involved in the study |
| --- | --- |
| ☒ | ☐ ChIP-seq |
| ☒ | ☐ Flow cytometry |
| ☒ | ☐ MRI-based neuroimaging |
